# Simulation of the Bell inequality violation based on quantum steering concept

**DOI:** 10.1038/s41598-021-84438-9

**Published:** 2021-03-11

**Authors:** Mohsen Ruzbehani

**Affiliations:** Photonics and Quantum Technologies Research School, Nuclear Science and Technology Research Institute, Tehran, Iran

**Keywords:** Quantum information, Quantum mechanics, Quantum simulation

## Abstract

Violation of Bell’s inequality in experiments shows that predictions of local realistic models disagree with those of quantum mechanics. However, despite the quantum mechanics formalism, there are debates on how does it happen in nature. In this paper by use of a model of polarizers that obeys the Malus’ law and quantum steering concept, i.e. superluminal influence of the states of entangled pairs to each other, simulation of phenomena is presented. The given model, as it is intended to be, is extremely simple without using mathematical formalism of quantum mechanics. However, the result completely agrees with prediction of quantum mechanics. Although it may seem trivial, this model can be applied to simulate the behavior of other not easy to analytically evaluate effects, such as deficiency of detectors and polarizers, different value of photons in each run and so on. For example, it is demonstrated, when detector efficiency is 83% the *S* factor of CHSH inequality will be 2, which completely agrees with famous detector efficiency limit calculated analytically. Also, it is shown in one-channel polarizers the polarization of absorbed photons, should change to the perpendicular of polarizer angle, at very end, to have perfect violation of the Bell inequality (2 $\sqrt 2$ ) otherwise maximum violation will be limited to (1.5 $\sqrt{2}$).

## Introduction

More than a half-century after celebrated Bell inequality^1^, nonlocality is almost totally accepted concept which has been proved by numerous experiments. Although there is no doubt about validity of the mathematical model proposed by Bell to examine the principle of the locality, it is comprehended that Bell’s inequality in its original form is not testable. Therefore, some other simpler to test experimentally forms of Bell’s inequality were proposed. Among them and most practical one is CHSH inequality^2^. As the validity of mathematically proof of Bell’s inequalities is accepted widely, all experiments reported so far required additional assumptions^3^. These additional assumptions constitute several famous loopholes, such as; “locality” loophole, “detector” or “fair-sampling” loophole, “freedom-of-choice” loophole, coincidence-time loophole^4–6^ or some obscure one as loophole of ‘individual existence’^7^. There are many reports regarding closing these loopholes^5,8–11^. Despite these experiments, although it is known entanglement is at the root of Bell’s theorem^12^, there are debates on how does Nature act so. In this article, based on a computer model, without using mathematical formulism of quantum mechanics, a simple picture of what may happen in nature is given. Although it may seem trivial, however we can see how this model can be applied to simulate some effects, such as photon absorption in one-channel polarizers and deficiency of detectors.

At first, to review the Bell’s inequality, consider a system with four observables: $A$, $A^{\prime}$ , $B$ and $B^{\prime}$ with possible values of $a$, $a^{\prime}$, $b$ and $b^{\prime}$ respectively, bounded by $\pm 1$. If we consider a hidden variable $\lambda$ with normalized distribution $\int {\rho (\lambda )d\lambda = 1}$, which can be decoupled the output of measurements, i.e.

1 $$p(ab|xy,\lambda ) = p(a|x,\lambda )p(b|y,\lambda ).$$

we can write the locality condition as:

2 $$p(a,b|x,y) = \int {\rho (\lambda )p(a|x,\lambda ).p(b|y,\lambda )d\lambda } .$$

in the case of validity of locality.

Based on this relation several inequalities, so named Bell’s inequalities, derived which give quantitative measure to check the validity of locality condition. As stated earlier, the most practical one is the CHSH inequality, used in this article.

To describe the practical method of testing CHSH, consider a source emitting entangled photon pairs such that the two-photon polarization state is expressed as:

3 $$\left| \psi \right\rangle = \frac{{\left| {x_{1} } \right\rangle \left| {x_{2} } \right\rangle + \left| {y_{1} } \right\rangle \left| {y_{2} } \right\rangle }}{\sqrt 2 },$$

where $\left| {x_{1} } \right\rangle$ and $\left| {y_{1} } \right\rangle$ represent photons propagating in -z direction with linear polarization, respectively in the x and y directions, with corresponding definition for photons propagating in z direction, i.e. $\left| {x_{2} } \right\rangle$ and $\left| {y_{2} } \right\rangle$.

**Figure 1:**
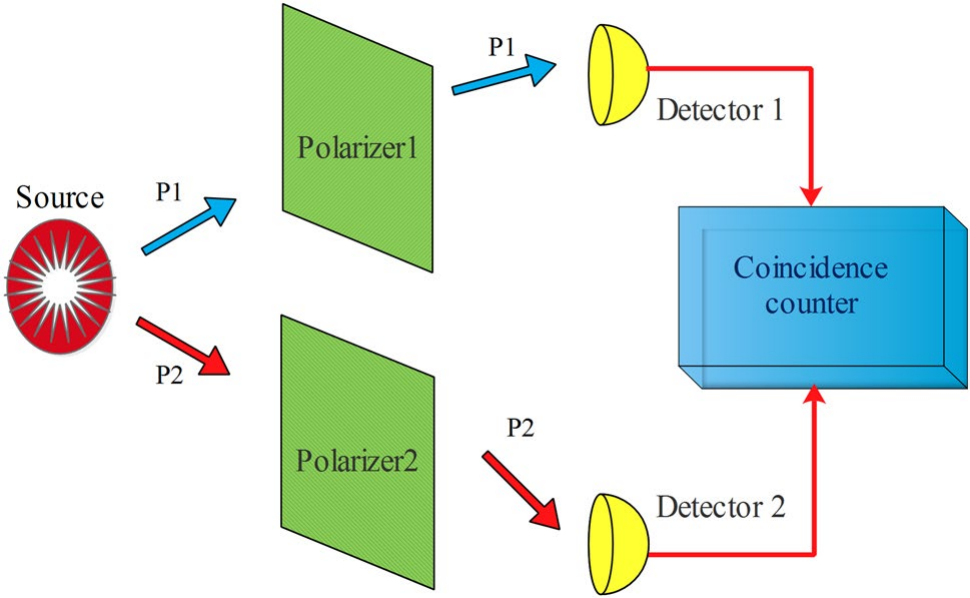
Diagram of a generic Bell test. A source emits pairs of entangled photons. Each photon after passing a one-channel polarizer, which its direction can be set by experimenter, is detected by a detector. Coincidence counter monitors coincidences due to a predefined interval.

Suppose we perform polarization measurement on photons by two one-channel polarizers, first one oriented in directions $(\alpha_{1} ,\alpha_{2} )$(Alice side) and second one $(\beta_{1} ,\beta_{2} )$(Bob side), followed by a detector. The result is 1 if a photon pass through or 0 if it is absorbed (stopped) by polarizers. Therefore, it is possible to consider a polarizer and its detector as a system that generates binary output due to passing or non-passing photons. In experiments based on the requirements formulated by Bell, measurement settings must be chosen randomly and independently by each party to close the freedom-of-choice loophole^9,13^. Furthermore, the locality loophole (or communication loophole) is open if the setting choice or the measurement result of one side could be communicated to the other side in time to influence the measurement result there. However, it is clear when we perform simulation, there is impossible that two simulated polarizers communicate with each other except if we have already made relation between them. Therefore, we can neglect freedom-of-choice loophole and perform the simulation for every pair of angles say *n* times and then collect the results, instead of doing 4*n* times for four randomly chosen angles pairs, as usually done in experiments. Also, in experiments with one channel polarizer the measurements of polarization are inherently incomplete because if no count is gained at a detector, there is impossible to recognize whether it is missed by the (low-efficiency) detector or it is blocked by the polarizer^14^. Therefore, in experiments it is necessary to perform tests with polarizers removed to compensate this problem. However, in simulation this is not the case because we can count zeros and a 0 always means that a photon has not passed. Therefore, by measuring coincidence counts, polarization correlation coefficient, for polarizers in orientations *α* and *β* can be defined as follows^15^:

4 $$E(\alpha ,\beta ) = \frac{{N_{11} (\alpha ,\beta ) + N_{00} (\alpha ,\beta ) - N_{10} (\alpha ,\beta ) - N_{01} (\alpha ,\beta )}}{{N_{11} (\alpha ,\beta ) + N_{00} (\alpha ,\beta ) + N_{10} (\alpha ,\beta ) + N_{01} (\alpha ,\beta )}}$$

in which e.g., $N_{10} (\alpha ,\beta )$ denotes the number of coincidence counts of 1 at first polarizer at angle of *α* and 0 at second polarizer at angle of *β* (or 1 at polarizer 2 at angle of $\beta^{ \bot } = \beta + 90^{o}$ as in practice) and so on. In Fig. 1, the basic scheme is sketched.

By performing four measurements of this type in orientations $(\alpha_{1} ,\beta_{1} )$,$(\alpha_{1} ,\beta_{2} )$ $(\alpha_{2} ,\beta_{1} )$ and $(\alpha_{2} ,\beta_{2} )$ one obtains the well-known Clauser-Horne-Shimony-Holt inequality (CHSH)^2^.

5 $$S(\alpha_{1} ,\alpha_{2} ,\beta_{1} ,\beta_{2} ) = E(\alpha_{1} ,\beta_{1} ) - E(\alpha_{1} ,\beta_{2} ) + E(\alpha_{2} ,\beta_{1} ) + E(\alpha_{2} ,\beta_{2} ) \le 2.$$

To check the difference of using two channel polarizers, let us find the quantum mechanical prediction of *S* parameter, when in each side there is an ideal two channel polarizer (polarizing beam splitter), each followed by two ideal detectors *D*_*ij*_(*i, j* = 1, 2). The transmission axes of the polarizers are set in the directions *a* and *b*, and the result of measurements can be considered 1 when there is a detection in *D*_*11*_ or *D*_*21*_ and -1 to detection in *D*_*I2*_ or *D*_*22*_. If we consider an operator $A(a)$ for the result of the measurement in -z direction, parallel and perpendicular to *a* with eigenvectors $\left| {a^{ \pm } } \right\rangle$ and eigenvalues $a = \pm 1$, and $B(b)$ for the result of the measurement in z direction, parallel and perpendicular to *b* with eigenvectors $\left| {b^{ \pm } } \right\rangle$ and eigenvalues $b = \pm 1$, by projection, following equations could be resulted^16^.

6 $$\begin{gathered} \left| {a^{ + } } \right\rangle = \cos \alpha \left| x \right\rangle_{1} + \sin \alpha \left| y \right\rangle_{1} , \, \left| {a^{ - } } \right\rangle = - \sin \alpha \left| x \right\rangle_{1} + \cos \alpha \left| y \right\rangle_{1} \hfill \\ \left| {b^{ + } } \right\rangle = \cos \beta \left| x \right\rangle_{2} + \sin \beta \left| y \right\rangle_{2} , \, \left| {b^{ - } } \right\rangle = - \sin \beta \left| x \right\rangle_{2} + \cos \beta \left| y \right\rangle_{2} \hfill \\ \end{gathered}$$

where $\left| x \right\rangle_{1}$ and $\left| y \right\rangle_{1}$ denote polarization states on the left (-z), $\left| x \right\rangle_{2}$ and $\left| y \right\rangle_{2}$ on the right (z), while *α* and *β* are the angles between the x axis and a and b, respectively. By definition it follows that

7 $$\begin{gathered} A(a) = + 1\left| {a^{ + } } \right\rangle \left\langle {a^{ + } } \right| + ( - 1)\left| {a^{ - } } \right\rangle \left\langle {a^{ - } } \right| \hfill \\ B(b) = + 1\left| {b^{ + } } \right\rangle \left\langle {b^{ + } } \right| + ( - 1)\left| {b^{ - } } \right\rangle \left\langle {b^{ - } } \right| \hfill \\ \end{gathered}$$

We can define the correlation coefficient $E(\alpha ,\beta )$ as follows.

8 $$\begin{gathered} E(\alpha ,\beta ) = \frac{{N_{ + + } (\alpha ,\beta ) + N_{ - - } (\alpha ,\beta ) - N_{ + - } (\alpha ,\beta ) - N_{ - + } (\alpha ,\beta )}}{{N_{ + + } (\alpha ,\beta ) + N_{ - - } (\alpha ,\beta ) + N_{ + - } (\alpha ,\beta ) + N_{ - + } (\alpha ,\beta )}} \hfill \\ { = }P_{ + + } (\alpha ,\beta ) + P_{ - - } (\alpha ,\beta ) - P_{ + - } (\alpha ,\beta ) - P_{ - + } (\alpha ,\beta ) \hfill \\ \end{gathered}$$

in which $N_{ + + } (\alpha ,\beta )$ denotes the number of coincidence counts of 1 at first polarizer at angle of *α* and -1 at second polarizer at angle of *β* and $P_{ + + } (\alpha ,\beta )$ denotes the probability of such coincidence and so on*.* If we calculate the expectation value of $A \otimes B$ it follows that

9 $$\begin{gathered} \left\langle \Psi \right|A \otimes B\left| \Psi \right\rangle = \left| {\left\langle {b^{ + } } \right|\left\langle {a^{ + } } \right|\left. \Psi \right\rangle } \right|^{2} + \left| {\left\langle {b^{ - } } \right|\left\langle {a^{ - } } \right|\left. \Psi \right\rangle } \right|^{2} - \left| {\left\langle {b^{ + } } \right|\left\langle {a^{ - } } \right|\left. \Psi \right\rangle } \right|^{2} - \left| {\left\langle {b^{ - } } \right|\left\langle {a^{ + } } \right|\left. \Psi \right\rangle } \right|^{2} \hfill \\ \, = P_{ + + } (a,b) + P_{ - - } (a,b) - P_{ - + } (a,b) - P_{ + - } (a,b) = E(a,b) \hfill \\ \end{gathered}$$

In which $\left| \Psi \right\rangle$ is the state vector of two photons as defined in (3). By substituting (3) and (7) in this equation if follows^16^

10 $$\begin{gathered} P_{ + + } (a,b) = P_{ - - } (a,b) = \frac{1}{2}\cos^{2} (\alpha - \beta ) \hfill \\ P_{ + - } (a,b) = P_{ - + } (a,b) = \frac{1}{2}\sin^{2} (\alpha - \beta ) \hfill \\ \end{gathered}$$

therefore

11 $$E_{QM} (a,b) = \cos 2(\alpha - \beta )$$

Finally, according to (5) and (11) $S_{QM} (\alpha_{1} ,\alpha_{2} ,\beta_{1} ,\beta_{2} )$ will be as follows

12 $$S_{QM} (\alpha_{1} ,\alpha_{2} ,\beta_{1} ,\beta_{2} ) = \cos 2(\alpha_{1} - \beta_{1} ) - \cos 2(\alpha_{1} - \beta_{2} ) + \cos 2(\alpha_{2} - \beta_{1} ) + \cos 2(\alpha_{2} - \beta_{2} )$$

which for particular set of orientation:

13 $$S_{QM - Max} = 2\sqrt 2$$

In practice when the number of photons tends to infinite, measured *S* value tends to this limit. It should be noted in ideal two-channel polarizers, on each polarizers side, always one of the detectors have a click on detectors. Therefore, we always “*measure*” the polarization of photons, despite the case in one-channel polarizers, in which we have no measurement when a photon absorbed.

## Results

It is known in an accurate experiment, artificial faking of Bell inequality even with very powerful computers is impossible^17^. This is the base of all attempts toward secure quantum communication. However, it does not mean modeling of the phenomena by computers also is impossible. As we know only a nonlocal model can generate result as quantum mechanics, however we will reach the final model step by step. Let us initially consider a local model in which a hidden variable (e.g., polarization of photons) and angle of polarizers are the variables. Suppose a source emits pairs of entangled photons so called the *signal* and the *idler* with polarization $\lambda_{ \, i}$ in different directions. Then an array of uniformly distributed random numbers over interval 0 and 2π as $\Lambda_{s} = [\lambda_{1s} ,...,\lambda_{is} ,...,\lambda_{ns} ]$ can represent sequence of emitted *signal* photons with random polarizations and $\Lambda_{I} = [\lambda_{1I} ,...,\lambda_{iI} ,...,\lambda_{nI} ]$ can represent the *idler*, which due to (3) just after emission before hitting photons to the polarizers we have $\Lambda_{I} = \Lambda_{s}$. Later we will see in a non-local model how the polarization of signal and idler will evolve. In this formalism we ignore time in equations and with this simplification, i*th* elements of two vectors are representing two entangled photons generated simultaneously. We can define the polarizer (and detector) function as $A(\alpha ,\lambda )$, in which *α* is the angle of polarizer and $\lambda$ is the polarization of the input photon. The output of this function is 1 or 0 due to passing or non-passing of the related photon. At this moment we assume perfect detectors with 100% efficiency, non-ideal case will be considered later. Therefore, there is no missed photon and any 1 represents a passed photon and any 0 corresponds to a stopped one. If we consider $\Lambda$ as input vector to a polarizer, output vector is a binary vector with same dimension as input vector. Although it may be argued, we can consider this model as a realistic one, because it is supposed that every photon before the incident has a defined polarization, thus its chance to pass through the polarizer is specified.

To obtain a perfect model, i.e., a model with similar behavior as nature, three rules should be considered. 1 – Polarizers model should obey the Malus’ law. 2—Symmetry property, i.e., if we consider a sequence of photons with random polarization uniformly distributed over {0, 2π} as input to the polarizer, fifty percent of them should pass for any angle setting of the polarizer. By these two rules our polarizer model acts similar to the realistic polarizers. 3—Finally and most crucial one, when we consider entangled photons as input of polarizers, output should be so that the correlation function obeys quantum mechanics correlation. To have a perfect model, all these conditions must be satisfied simultaneously. To fulfil first and second criteria, following model of polarizer is sufficient. If we define input vector as $\Lambda$ with *n* elements, polarizer function can be defined as follows:

14 $$\begin{aligned} & A = Polarizer(\alpha ,\Lambda )\,\,{\text{in}}\,\,{\text{which}}\,\,\Lambda = [\lambda _{1} , \ldots ,\lambda _{i} , \ldots ,\lambda _{n} ]\quad for\quad i = 1:n \\ & t = (\cos (\alpha - \lambda _{i} ))^{2} \\ & {\text{if }}r \le t \Rightarrow A(\alpha ,\lambda _{i} ) = 1 \\ & {\text{if }}r > t \Rightarrow A(\alpha ,\lambda _{i} ) = 0, \\ \end{aligned}$$

in which *r* is a random number with uniform distribution over {0, 1}, and other parameters are as defined previously. Because in this article Matlab pseudo-random number generator was used, which we know its result is not true random numbers, the accuracy of our results may be questioned. However, because correlation of elements of this array to each other is not matter of simulation, randomness of them is not important. In fact, this array should be only uniformly distributed over the interval, no matter how the sequence is arranged. Because we check correlation of *signal* elements to those of *idler*. Moreover, it should be noted that the length of the vector is very short compared to the period of random number generated by Matlab, so it can not affect the accuracy of our results. In this model there is a random number *r* that generate indeterministic result. In other word, as it occurs in experiment, despite same polarization of two photons, one may pass and other may stopped by polarizer. However, because some factors such as angle and location of strike of photons to the polarizers are not same for photons with same polarization, it can be said, this kind of indeterminism arise from our ignorance.

**Figure 2:**
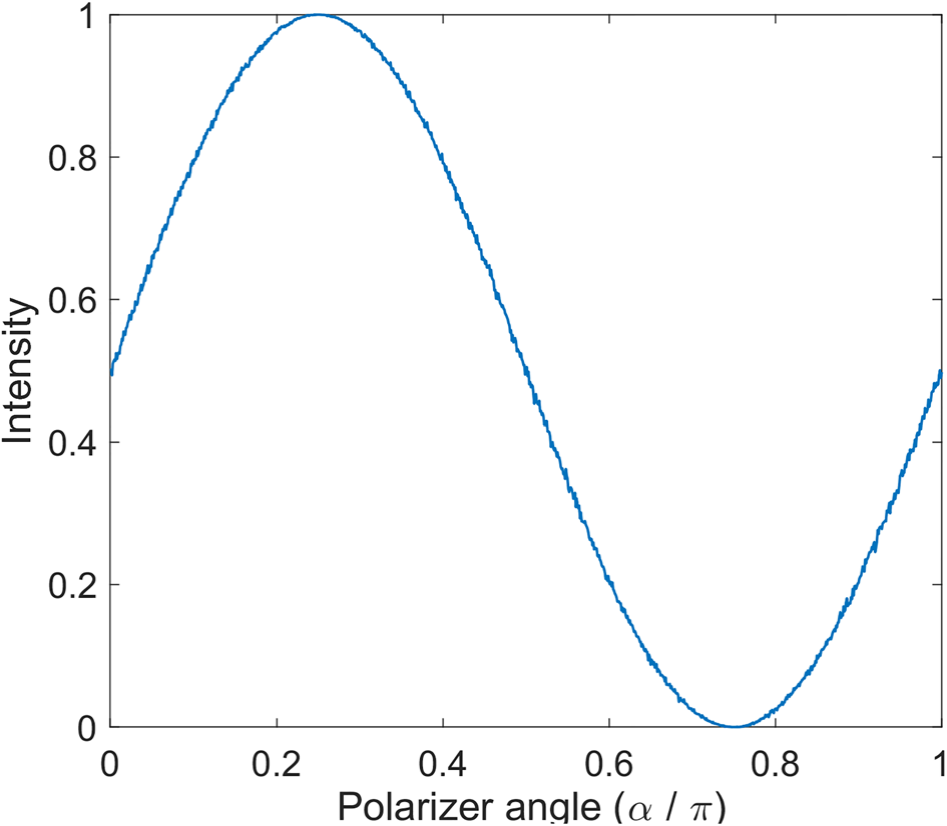
Malus’ law. The figure shows that how the presented model of polarizer obeys the Malus’ law. Y axis represents percentage of passed photons. In this figure polarizations of all input photons are π/4.

In (14) *t* is the probability of passing a photon through the polarizer. Clearly applying this rule to an ensemble of photons result in same intensity relation as Malus’ law. To check, we can consider a sequence of photons with same polarization. i.e.,$\lambda_{i} = \lambda_{c} {\text{ for }}i = 1:n$. By change of angle of polarizer and counting passed photons (ones of the output vector) a diagram that shows the percentage of passing photons, as illustrated in Fig. 2, is obtained, which completely agree with Malus’ law. In this case, polarizations of all input photons are chosen to be $\pi /4$. In all simulations in this article, Matlab program was used.

**Figure 3:**
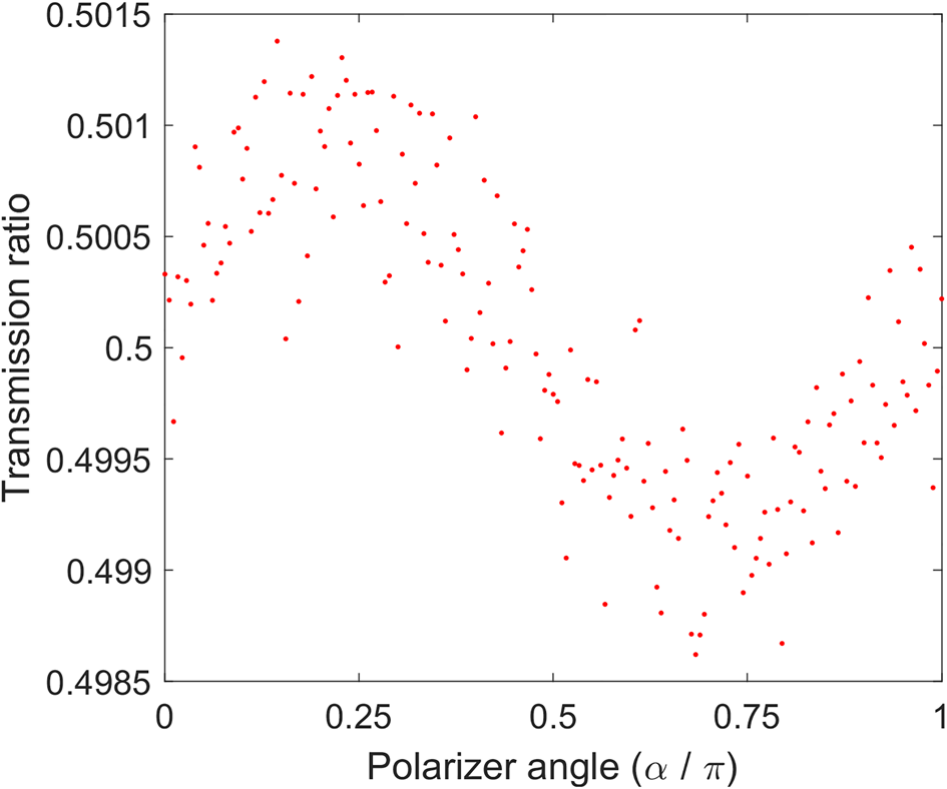
Symmetry condition satisfied by the presented polarizer model. The figure shows that in the proposed model, if N photons which their polarization distribution varies uniformly between 0 to 2π hit the polarizer, almost half of them will pass, independent of the polarizer angle setting. By increasing N this value tends to 0.5. In this example N = 1,000,000 and polarizer angle varies linearly between 0 to 2π. Every dot representing transmission ratio of passed photons in that angle.

In Fig. 3, second rule; symmetry property, was examined. It is seen, similar to the practical case, when a source emits photons with polarizations uniformly distributed over [0, 2π], for any polarizer angle setting, almost half of the photons pass through the polarizer.

To examine the third criterion, consider $\Lambda$ as input of *A* polarizer and because of (3) each photon has same polarization as its twin, for first step we consider same vector as input to *B* polarizer. It means at first step there is no steering and therefore, we have:

15 $$\begin{aligned} & \Lambda = [\lambda _{1} ,\lambda _{2} , \ldots ,\lambda _{n} ] \\ & A(\alpha _{1} ,\Lambda ) = Polarizer(\alpha _{1} ,\Lambda ) \\ & B(\beta _{1} ,\Lambda ) = Polarizer(\beta _{1} ,\Lambda ). \\ \end{aligned}$$

Because $A$ and $B$ are binary vectors we can define $A_{not} = 1 - A,$ and $B_{not} = 1 - B$. Then according to (4)

16 $$E(\alpha ,\beta ) = \frac{{sum\left( {A(\alpha ) \odot B(\beta )} \right) + sum\left( {A_{not} (\alpha ) \odot B_{not} (\beta )} \right) - sum\left( {A(\alpha ) \odot B_{not} (\beta )} \right) - sum\left( {A_{not} (\alpha ) \odot B(\beta )} \right)}}{{sum\left( {A(\alpha ) \odot B(\beta )} \right) + sum\left( {A_{not} (\alpha ) \odot B_{not} (\beta )} \right) + sum\left( {A(\alpha ) \odot B_{not} (\beta )} \right) + sum\left( {A_{not} (\alpha ) \odot B(\beta )} \right)}}$$

in which $\odot$ represents Hadamard product of vectors, e.g., $A \odot B = [a_{1} b_{1} ,...,a_{n} b_{n} ]$, that result in a vector in which every 1 corresponds to a coincidence detection. Therefore, total coincidence detection of photons could be found with *sum* operation (summation). In practice, instead of $A_{not} (\alpha )\left( {B_{not} (\beta )} \right)$, $A(\alpha^{ \bot } )\left( {B(\beta^{ \bot } )} \right)$ should be used, however in simulations because we can count zeros it is unnecessary to do that.

Until now we have considered a local realistic model. Now let to check the correlation function. If we let *α* and *β* vary in range [0 2π] and find $E(\alpha ,\beta )$ according to (16), its shape is a sinusoidal function of $(\alpha - \beta )$ as follows:

17 $$E(\alpha ,\beta ) = E_{0} \cos (2(\alpha - \beta )),$$

in which $E_{0}$ is its amplitude.

**Figure 4:**
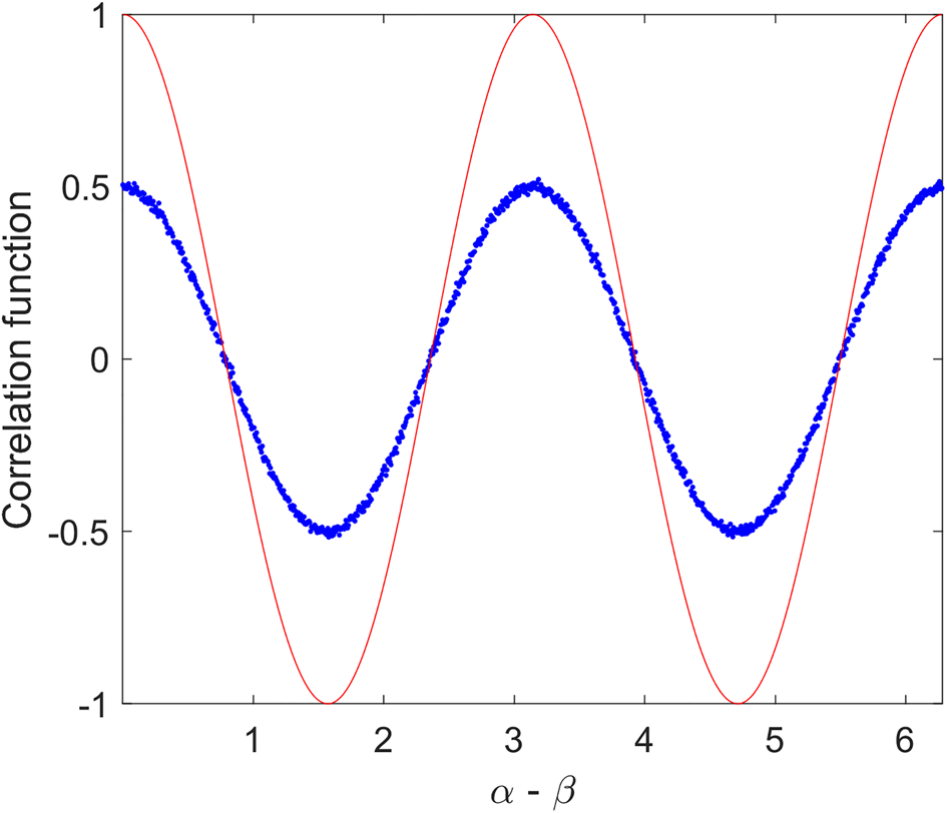
Simulation result of correlation function versus angle difference of polarizers based on relation (15) (dot line-blue) compared to quantum mechanics prediction (solid line-red).

**Figure 5:**
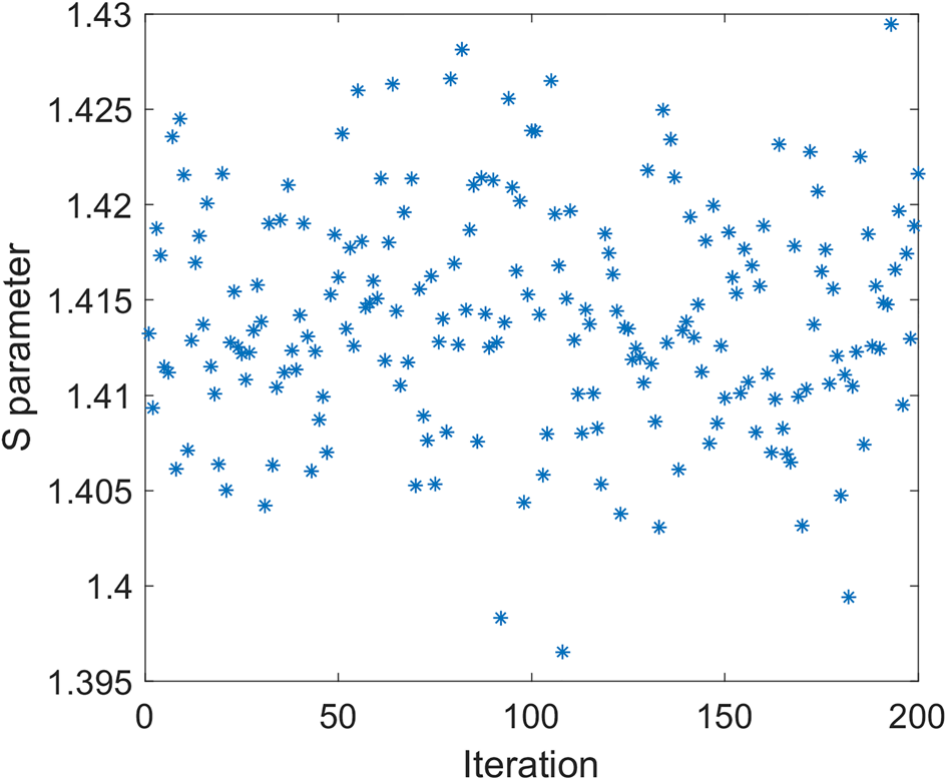
*S* parameter for 200 iterations for the local model. In each run input to each polarizer is a vector with size 100,000. Based on (15), *signa*l and *idler* vectors are equal. Mean value of S parameter in this example is equal to 1.414 while angles of polarizers are $\alpha_{1} = 3\pi /8{, }\beta_{1} = \pi /4, \, \alpha_{2} { = }\pi {/8, }\beta_{2} = 0.$

In Fig. 4, simulated correlation function for above local model is given. As it is seen in this figure, despite sinusoidal shape of the correlation function, its magnitude is half of that of quantum mechanics prediction, i.e., $E_{0} = 0.5$. In fact, violation of Bell’s inequality in quantum mechanics is due to the sinusoidal correlation relation with unity magnitude. Therefore, if one could find a model that shows such correlation, the result would be violation of Bell’s inequality. For the above model, it is easy to show the maximum value of *S* (Eq. 5) tends to $\sqrt 2$ as found in simulation. In Fig. 5, result of simulation for 200 runs has been given. In this case mean value of *S* parameter is equal to 1.414.

How is it possible to redefine the model to mimic the Nature? Evidently, with a local model it is not possible, so we should turn to a nonlocal one. For first step suppose that setting of each polarizer has effect on other polarizer output, i.e.,

18 $$\begin{gathered} A \equiv A(\alpha ,\beta ,\Lambda ) \hfill \\ B \equiv B(\alpha ,\beta ,\Lambda ). \hfill \\ \end{gathered}$$

This formalism led to some peculiar assumption as “conspiracy of the polarizers”^17^. However, there is another more rational way to realize similar dependency as follows.

19 $$\begin{gathered} A \equiv A(\alpha ,\Lambda_{1} ) \hfill \\ B \equiv B(\beta ,\Lambda_{2} ){ ,} \hfill \\ \end{gathered}$$

in which

20 $$\begin{gathered} \Lambda_{1} = \Lambda (\lambda ) \hfill \\ \Lambda_{2} = \Lambda (\lambda ,\alpha ). \hfill \\ \end{gathered}$$

In these relations, it is supposed the polarizer *A* (Alice side) receive the photon sooner than that of polarizer *B* (Bob side), related to the framework preferred by nature. Furthermore, it is not important which polarizer receive the photons sooner. What is important is this fact that both photons do not reach polarizers simultaneously (from preferred reference framework point of view). Even, if in an experiment we move polarizers toward and outward each other randomly, so that by chance *A* or *B* first receive photons sooner, it has no effect on overall correlation function^18^. Therefore, at each time when a photon from first group strikes, for example, polarizer *A* (*B*), its twin is in flight. Therefore, idler photons (signal photons) polarizations affected by *A* (*B*) polarizer setting. It is clear hereafter $\Lambda_{1}$ and $\Lambda_{2}$ are not hidden variables as stated by EPR, because it is supposed there is a superluminal connection between them. In first attempt, suppose because polarizations of passing photons through a polarizer change to the angle of that polarizer, polarizations of twin photons ($\Lambda_{2}$) change correspondingly. We do not know whether the change of polarization of twins is immediately or not? However it is clear, based on experiments, it should happen with speed larger than $D_{A - B} /(t_{1} - t_{0} )$, in which $D_{A - B}$ is spatial separation of polarizers and $t_{0}$, $t_{1}$ are time of hitting of a photon to polarizer *A* and its twin to *B* respectively (reference framework). In other words, if difference between source to polarizers distances is equal to *δ*, this speed is larger than $(D_{A - B} /\delta ) \times C$ in which *C* is the speed of light. In practice because of the non-ideality of polarizers and sources and reference frame relative speed, a correction factor must be added^19^. How does nature do it? We do not know, we may in agreement with Gisin say, “No story in space–time can tell us how nonlocal correlations happen; hence, nonlocal quantum correlations seem to emerge, somehow, from outside space–time”^20^.

From (19) and (20) we can infer following relations.

21 $$\begin{aligned} A(\alpha ,\Lambda _{1} ) & \equiv A(\alpha ,\Lambda ) \\ B(\beta ,\Lambda _{2} ) & \equiv B(\alpha ,\beta ,\Lambda ). \\ \end{aligned}$$

With this assumption it is possible to redefine the model, i.e., instead of considering same vectors as input of polarizers, we can change polarization of twins of those photons that pass through the polarizer *A* to α, and keep the polarization of twins of absorbed photons unchanged. It means, it is supposed that because absorbed photons’ polarization did not change, for first attempt, we keep their twins’ polarization unchanged. This matter is important because when we consider two-channel polarizers, each photon pass through polarizer, up or down, after strike to the polarizer. Therefore, every photon has a specific polarization after polarizer. However, this is not completely same as in one-channel polarizers and one may argue about polarization of absorbed photons, which have not been measured. Therefore, input vector of second polarizers (*B*) is as follows.

22 $$\Lambda_{2} = \alpha A + A_{not} \odot \Lambda .$$

where *α* is the *A* polarizer angle. First term in above relation corresponds to passed photons through polarizer *A* and second term corresponds to absorbed ones.

**Figure 6:**
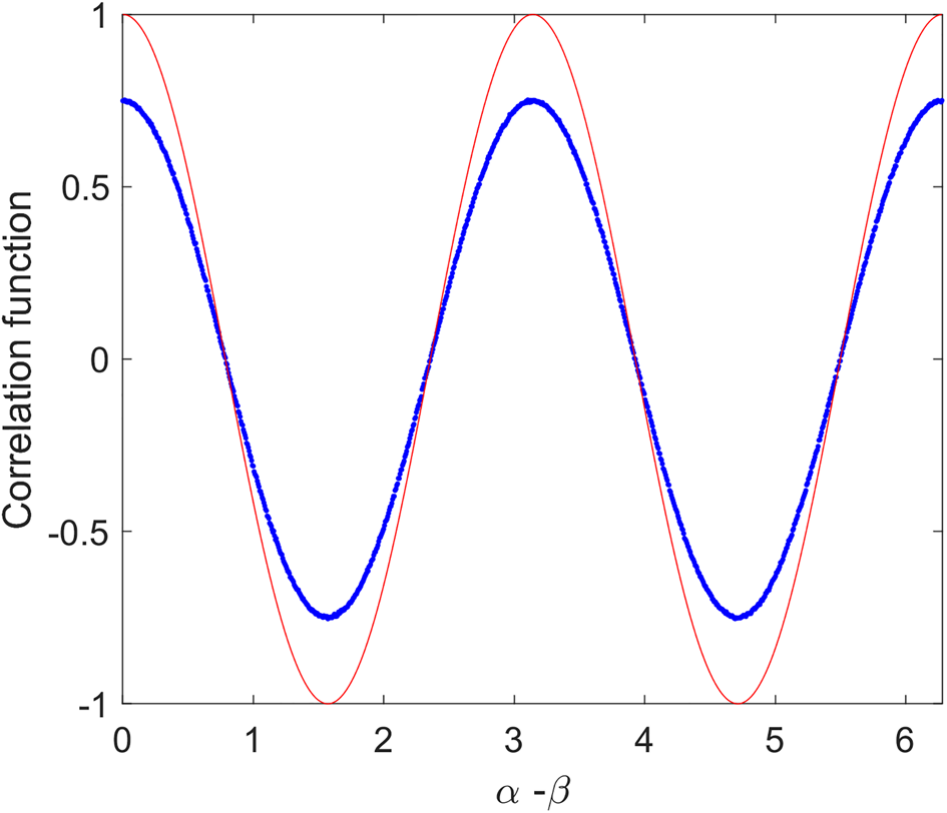
Simulation result of correlation function versus angle difference of polarizers based on relations (21) and (22) (dot line-blue) compared to quantum mechanics prediction (solid line-red).

**Figure 7:**
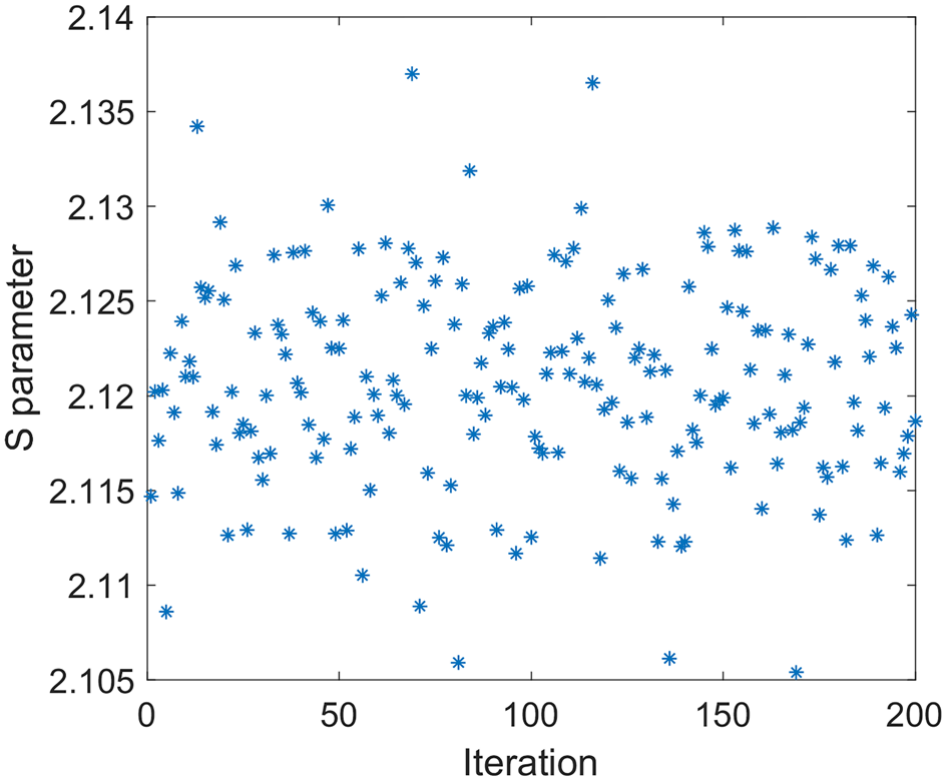
S parameter for 200 runs. In each run size of the input vector is 100,000. Polarization of photons that hit B polarizer obeys (22). Mean value of calculated S parameter in this example is equal to 2.121 while angles of polarizers are $\alpha_{1} = 3\pi /8{, }\beta_{1} = \pi /4, \, \alpha_{2} { = }\pi {/8, }\beta_{2} = 0$.

In Fig. 6, simulation of the correlation function for above case is given. It is shown, similar relation as (17) is valid. In this case by increasing number of photons, i.e., $n \to \infty$, $E_{0}$ tends to 0.75. This correlation function violates CHSH inequality by $1.5\sqrt 2$. In Fig. 7, result of simulation for 200 runs is shown. In this case mean value of *S* parameter is equal to 2.121.

Despite violation of Bell’s inequality with this assumption, the result doesn’t agree with quantum mechanics and also experiments. Until now we have found a nonlocal model that violate the CHSH inequality albeit, less than quantum mechanics prediction. It seems further modification is necessary. Let us redefine $\Lambda_{2}$ as follows.

23 $$\Lambda_{2} = \alpha A + (\alpha + \pi /2)A_{not} .$$

**Figure 8:**
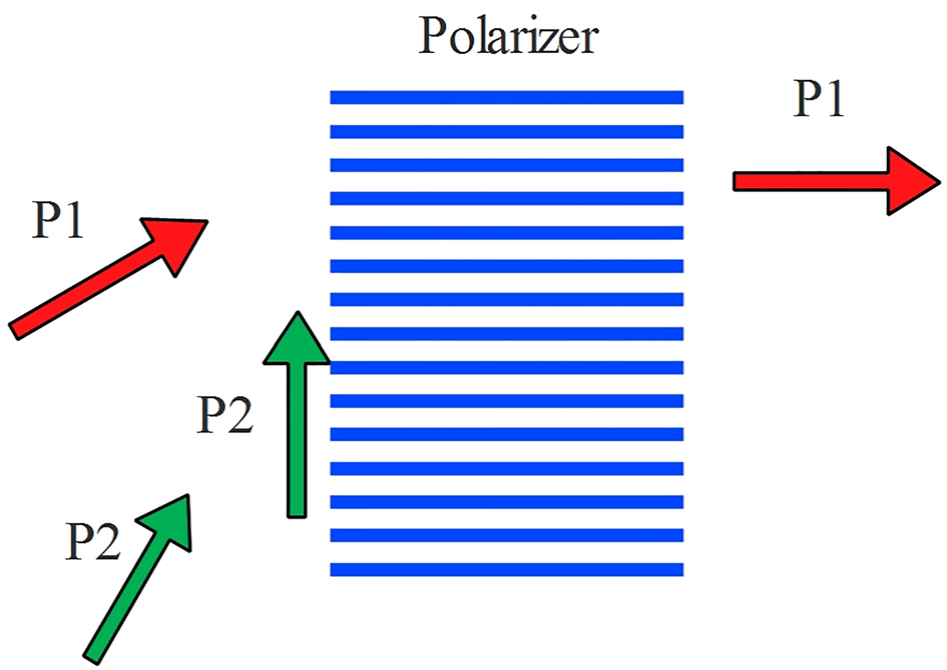
A naïve picture of what may happen in hitting photons to a polarizer. Chance of passing a photon through a polarizer is higher if its angle of polarization is closer to the polarization angle of the polarizer (Malus’ law). After passing a photon its polarization will change to the polarizer angle. If it doesn’t pass, its polarization (at the very end, before absorption) will change to the perpendicular to the polarization angle of the polarizer.

This relation means that polarization of photons which pass through the polarizer *A* are changed to the angle of polarizer and those that absorbed (stopped), at very end, become perpendicular to that. In Fig. 8, a naive picture of what may happen in collision of photons to a polarizer, is given. If we accept this matter, twins of these photons that hit second polarizer can be expressed by (23).

**Figure 9:**
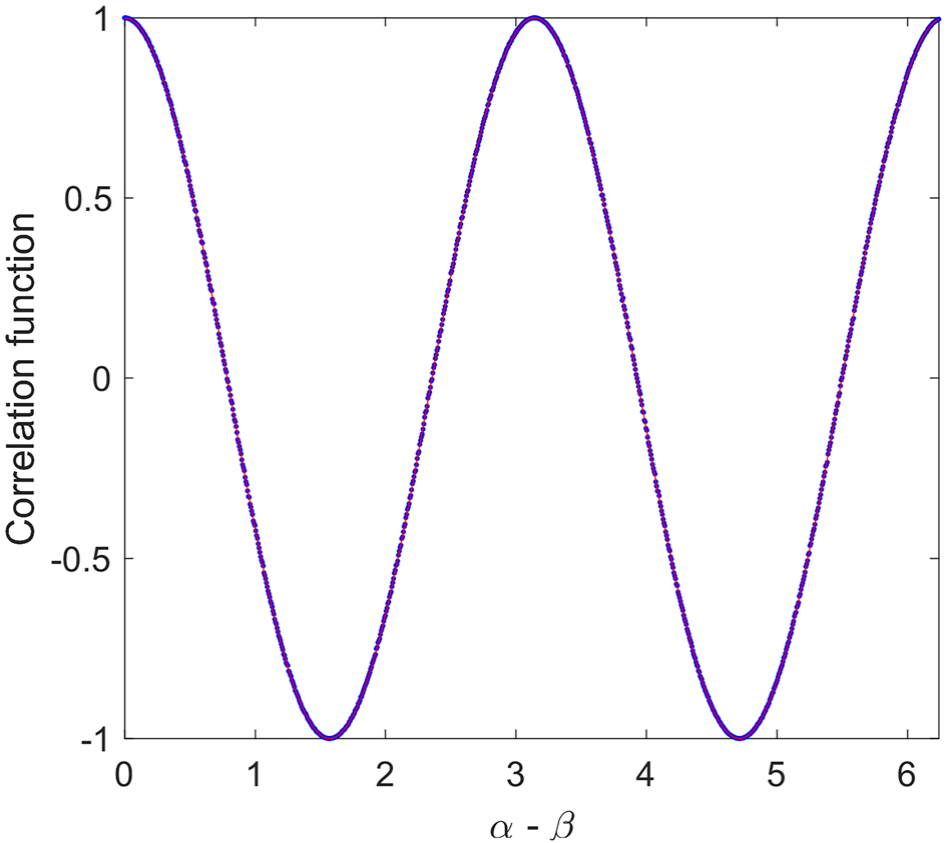
Simulation result of the correlation function. In this case polarizations of photons that hit second polarizer (B) are according to Eq. (23). As it is seen this correlation function completely agrees with quantum mechanics prediction.

Now, if we examine (23) we will get the desired result. In Fig. 9, simulation result of correlation function due to such relation is shown. It can be seen; the result completely agrees with quantum mechanics.

**Figure 10:**
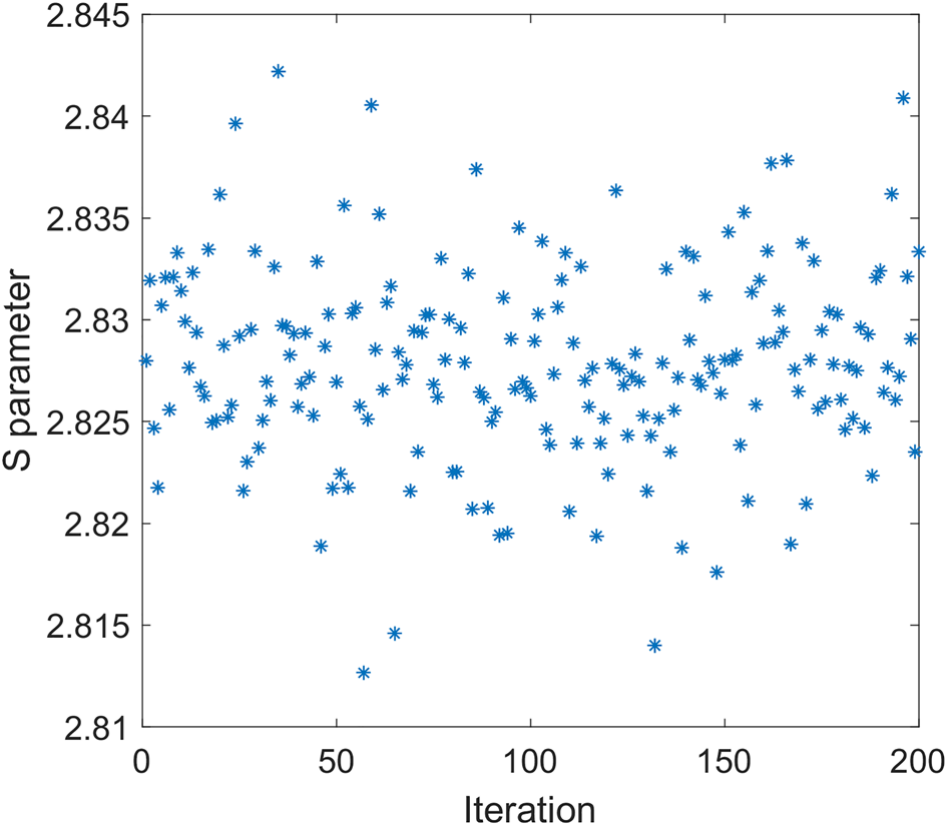
*S* parameter for 200 runs. In each run number of photons that strike each polarizer is 100,000. Polarization of photons that hit B polarizer obey (23). Mean value of S parameter in this example is equal to 2.828 while angles of polarizers are $\alpha_{1} = 3\pi /8{, }\beta_{1} = \pi /4, \, \alpha_{2} { = }\pi {/8, }\beta_{2} = 0$.

Clearly, such perfect correlation function violates CHSH ineqaulity as quantum mechanics by $2\sqrt 2$. In fact, perfect sinusoidal correlation is the key point of violation of the Bell inequality. It should be noted all efforts to find a way to violate Bell inequality with local models fail, because such perfect correlation could not be obtained by local models. On the other hand, the cause of such kind of correlation is not only superluminal connection of entangled photons, but also function of polarizers known as Malus’ law. If for example polarizers was so that only photons with polarization exactly same as their angle of polarization could pass through, the Bell inequality, would not be violated. The Cirel’son bound also is the result of function of polarizers. In Fig. 10, result of simulation for 200 runs is given. In this case mean value of *S* parameter is equal to 2.828.

It can be concluded if we accept that polarization of non-passing photons changes to perpendicular to the angle of polarizer, the perfect violation of Bell’s inequality occurs. As we know Cirel’son bound for the CHSH inequality is $2\sqrt 2$^21^ and there are arguments on whether stronger violation is possible in experiments or not^22^. According to the presented model and due to the polarizers function, it is clear why it is impossible to pass this limit in experiments.

**Figure 11:**
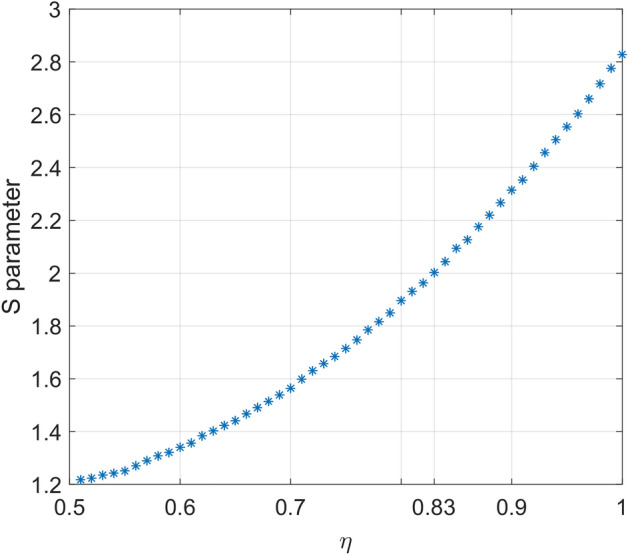
Effect of non-unity value of detectors efficiency on the *S* parameter. It is seen when detector efficiency in this example is 0.83, mean value of S parameter is about 2. In this example each star corresponds to the mean value of 50 runs, in each run 5000 photons considered with defined detector efficiency, while other parameters are same as those of Fig. 10.I

Another surprising result of this model is the simplicity of modeling other factors such as detector deficiency. Until now we have considered perfect detectors in our simulations. Due to the detector efficiency loophole, in case of non-unity efficiency of detectors, even local models may result in violation of the Bell inequality. Furthermore, the Bell inequality could not be violated with low efficient detectors. The method is simple, if a random variable with uniform distribution over {0, 1}, is less than detector efficiency (*η*), and in same time output of polarizer function is 1, the detector output will set to one, otherwise to zero. Therefore (1- *η*) percentage of ones transform to zeros. Consequently, correlation function should be reduced compared to ideal detector. In Fig. 11, detectors efficiency has varied between 0.5 to 1 and consequently *S* between 1.2 to 2.88. As can be seen, for detectors efficiency of 0.83 the *S* value is about 2, means no violation would happen for detector efficiency less than 0.83. This matter agrees with work of A. Garg et.al^23^, clearly approve the accuracy of the model. Although we did not consider dark counts, it is easy to evaluate this factor by changing zeros to ones in similar manner.

## Conclusion

In this article a nonlocal realistic model based on quantum steering concept with a simple model of polarizer which obeys the Malus’ law is given. This model simulates violation of the Bell’s inequality in experiments. Realistic model means that every measurable variable, in this case polarization of photons, exists regardless of our measurement. In proposed model polarization of every photon passing through the polarizer changes to the polarizer angle and consequently that of its twin changes quickly. Furthermore, it is shown by simulation, in one-channel polarizers the polarization of absorbed photons should change to the perpendicular of polarizer angle, at very end, to have perfect violation of the Bell inequality (2$\sqrt 2$) otherwise maximum violation will be limited to 1.5$\sqrt 2$. Although, in this article one-channel polarizers were considered in simulations, it could be extended to two-channel polarizers easily.

Furthermore, effect of non-unity value of detectors efficiency was examined. In fact, main benefit of the presented method is to simulate some other phenomena such as maximum attainable violation based on given detectors and or polarizers efficiency, dark counts, different number of photons in each run and so on. Simply, this method of simulation may provide an easy method of examination of some not easy to setup test without dealing with complicated analytical solution.
