# Author Correction: Simulation of the Bell inequality violation based on quantum steering concept

**DOI:** 10.1038/s41598-021-91692-4

**Published:** 2021-06-10

**Authors:** Mohsen Ruzbehani

**Affiliations:** Photonics and Quantum Technologies Research School, Nuclear Science and Technology Research Institute, Tehran, Iran

Correction to: *Scientific Reports* https://www.doi.org/10.1038/s41598-021-84438-9, published online 11 March 2021

The original version of this Article contained an error in Reference 11, which was incorrectly given as:

Mitchell, M. W. *et al.* Device-independent randomness expansion with entangled photons. *Nature (London)* **557**, 212 (2018).

The correct reference is listed below:

BIG Bell Test Collaboration., Abellán, C., Acín, A. *et al.* Challenging local realism with human choices. *Nature* **557**, 212–216 (2018).

The original Article has been corrected.

